# The welfare problems of wide-ranging Carnivora reflect naturally itinerant lifestyles

**DOI:** 10.1098/rsos.230437

**Published:** 2023-09-06

**Authors:** Miranda Bandeli, Emma L. Mellor, Jeanette Kroshko, Hafiz Maherali, Georgia J. Mason

**Affiliations:** ^1^ Department of Animal Biosciences, University of Guelph, Ontario, Canada; ^2^ Department of Integrative Biology, University of Guelph, Ontario, Canada; ^3^ Bristol Veterinary School, University of Bristol, Bristol, UK

**Keywords:** welfare, captivity, zoos, choice, control, Carnivora

## Abstract

Carnivora with naturally small home ranges readily adjust to the evolutionarily new environment of captivity, but wider-ranging species seem prone to stress. Understanding why would advance both collection planning and enclosure design. We therefore investigated which aspects of wide-ranging lifestyles are key. We identified eight correlates of home range size (reflecting energetic needs, movement, intra-specific interactions, and itinerant lifestyles). We systematically assessed whether these correlates predict welfare better than range size *per se*, using data on captive juvenile mortality (from 13 518 individuals across 42 species) and stereotypic route-tracing (456 individuals, 27 species). Naturally itinerant lifestyles (quantified via ratios of daily to annual travel distances) were found to confer risk, predicting greater captive juvenile losses and stereotypic time-budgets. This finding advances our understanding of the evolutionary basis for welfare problems in captive Carnivora, helping explain why naturally sedentary species (e.g. American mink) may breed even in intensive farm conditions, while others (e.g. polar bears, giant pandas) can struggle even in modern zoos and conservation breeding centres. Naturally itinerant lifestyles involve decision-making, and strategic shifts between locations, suggesting that supplying more novelty, cognitive challenge and/or opportunities for control will be effective ways to meet these animals' welfare needs in captivity.

## 1. Introduction

For wild animals, captivity is an evolutionarily new environment to which some species adjust readily, but others respond with signs of stress, such as health, reproductive and behavioural problems (e.g. [1,2]). Such inter-species variation has been evident since the dawn of domestication (when some species adjusted well to management by humans, but others did not; e.g. [3,4]); and it can still be seen today, in modern zoos, breeding centres and aviaries. Here, recorded data from multiple species can enable the use of phylogenetic comparative methods to identify aspects of natural behavioural biology that pre-adapt some animals to thrive in captive conditions, while placing others at risk. Results of such analyses can then guide management decisions (e.g. suggesting new ways to reduce mismatches between wild and captive environments), and also yield fundamental new ‘evo-mecho’ insights into the motivational control of behaviour (e.g. [1,5]). The first such research focused on the Carnivora: a charismatic order in which 27% of its 285 extant species are Threatened [6], and many live in captive conditions (ranging from safari parks to more restrictive fur/musk/traditional Chinese medicine farms; e.g. [7]). Three hypothesized welfare risk factors were investigated in this work [8,9]—being naturally territorial, wide-ranging or reliant on predation—using two stress-sensitive, management-relevant response variables: stereotypic behaviour and elevated infant mortality. Stereotypic behaviour indicates poor welfare (e.g. [10–12]). It is also abnormal (e.g. [13]), disliked by the public [14] and a potential obstacle to captive breeding [15]. In Carnivora, it typically involves repetitive route-tracing (e.g. pacing), which potentially originates from escape attempts [16]. Elevated infant mortality can indicate maternal stress (reviewed in [17,18]); and it can also compromise population sustainability (e.g. [19,20]). For both of these variables, having naturally large annual home ranges (AHRs) emerged as the key risk factor, so contradicting long-standing beliefs that stereotypic route-tracing reflects frustrated motivations to hunt (e.g. [21]).

Over the next two decades, this finding helped to inspire some creative new enclosure designs for Carnivora (e.g. [22,23]). Phylogenetic comparative methods were also subsequently used to identify behavioural, cognitive and dietary risk factors in other taxa (namely captive Primates [24]; Ungulates [5,25–27]; Parrots [28]). Furthermore, comparative research on captive Carnivora continued. San Diego Zoo and Safari Park researchers found a trend for AHR to predict pacing in their collections, and new evidence of poor captive breeding: naturally wide-ranging species produced fewer live young per mated female [12]. In parallel, we updated the Clubb & Mason database with 10 more years of behavioural data (2000–2010; [29]), before running new analyses that replicated the AHR effect on route-tracing, but failed to do so for infant mortality [29,30].

Here, we build on this corpus of work to solve two outstanding problems. First, despite the reliable relationships seen between AHR and route-tracing, these effects were never strong (*R*^2^ < 30%); and similar issues applied to the more inconsistent reproductive correlates of AHR size [12,30]. This greatly limited the value of AHR size for predicting Carnivore welfare. Second, having a large AHR is not an isolated attribute: wide-ranging species differ from little-ranging ones in multiple ways. This made it very uncertain how best to improve captive care for wide-ranging Carnivora, with different authors drawing disparate conclusions (for example, that wide-ranging Carnivora need more space [31–33], more freedom to locomote (e.g. [34]) or instead more opportunities for choice or control (e.g. [35])). To tackle these problems, here we therefore updated our route-tracing dataset with another 5 years of behavioural studies (2011–2015; [36,37]), and replaced our dated zoo infant mortality data (spanning 1988–2000) with more contemporary values [38], before testing a series of new hypotheses that aimed to pinpoint the *specific attributes* of wide-ranging lifestyles that more strongly predict captive welfare.

The present work thus aims to assess whether AHR size *per se* is the most relevant welfare risk factor for captive Carnivora, or whether a correlated attribute is instead the key. Large AHRs arise from many interconnected drivers, including trophic level and large body sizes [39,40]. Furthermore, large AHRs have consequences, some arising from the resulting itinerant, ‘semi-nomadic’ lifestyles in which animals shift between spatially separated regions of their extensive ranges [17,41–43]. Such lifestyles involve exploring novel environments, and creating multiple dens or resting places annually [17,30,42,44], as well as using navigation skills, perhaps aided by neurological adaptations for spatial cognition [42,45]. *Any* of these correlated attributes could therefore underlie the apparent effect of AHR size on Carnivora welfare. Here, we therefore systematically investigate a range of correlates of AHR size, to see if any of these are better predictors of welfare than is AHR size itself.

Why pursue these questions? Because welfare and management problems remain, even in modern zoos with improved animal care (cf. e.g. [38]). In some species, polar bears (*Ursus maritimus*) being a prime example (e.g. [13]), route-tracing is still prevalent and often time-consuming ([e.g. [46–49]), even in contemporary conservation breeding centres and thoughtfully designed zoo enclosures (e.g. [12,50,51]). Furthermore, once stereotypic behaviour has developed, it becomes hard to eliminate without pharmacological treatments like fluoxetine (‘Prozac'; e.g. [52–55]). A deeper understanding of the aetiology of stereotypic behaviour is therefore needed, to both better anticipate its emergence before it appears, *and* to improve chances of eliminating it via improved housing and husbandry [52,56]. Furthermore, high mortality in young animals is also still evident in some Carnivora species. In African wild dogs (*Lycaon pictus*), for example, some 75% of zoo-born offspring fail to reach adulthood [57], with poor offspring survival also reported for contemporary zoo-housed polar bears [58], Asiatic lions, (*Panthera leo persica*) [59], fishing cats (*Prionailurus viverrinus*) [19], maned wolves, (*Chrysocyon brachyurus*) [20] and giant otters (*Pteronura brasiliensis*) [38]. This is worrying because most zoo-based managed breeding programmes are not self-sustaining [51], and most global captive population sizes are small (the median for zoo-housed Carnivora being 30 individuals [60]). This makes it important to identify the fundamental needs of Carnivora, if even naturally wide-ranging species like polar bears are ever to achieve low stress, behaviourally normal, self-sustaining captive populations.

## 2. Methods

### 2.1. Welfare-sensitive outcome variable data collation

#### 2.1.1. Updating our database on stereotypic route-tracing

To our route-tracing database, we added data from literature published 2011–2016 (see electronic supplementary material for details), increasing the number of species with stereotypic behaviour time-budgets to 56. The final sample for analyses (after excluding species with less than five subjects) was 27 species, representing 456 route-tracing individuals (see electronic supplementary material, table S1).

From the same papers, we also extracted data on husbandry, in order to update two variables identified as likely potential confounds (since species systematically vary in how typically housed): species-typical degree of cover in the enclosure, and feeding enrichment provision (with electronic supplementary material, tables S2 and S3 providing details).

#### 2.1.2. Captive juvenile mortality database

Values for births in *Species360* member zoos dying before 366 days of age were extracted for the period 2010–2019, representing 13 518 births across 42 of our 56 species (extracted from [38]; see electronic supplementary material for details). These values span infant and juvenile periods, but are termed ‘juvenile mortality’ for brevity. To control for species differences in life-history and reproductive strategy that represent intrinsic influences on offspring mortality (cf. [61,62]), a potential source of noise, we also quantified the degree to which offspring are altricial versus precocial via the ages at which infants' eyes open (from [63]). Electronic supplementary material, table S1 gives the final species values.

#### 2.1.3. Collating data on ranging and its correlates

The ecological literature identified 12 potential correlates of AHR size for which data were also available across Carnivora. These comprised nine drivers or selection pressures likely to influence AHR size, and three likely biological consequences of being wide-ranging. Their rationales, and associated predictions, are as follows.

First, three inter-related potential drivers of large AHRs were body mass, individual metabolic need, and group metabolic need. Large body sizes and high metabolic demands involve high energetic requirements which favour large AHRs [40,64–66]. For social animals, larger groups of larger animals also need larger AHRs to meet collective energetic requirements [40]. If such factors underlie AHR's role in captive welfare, then species with larger body masses, or greater individual/group metabolic needs, will perform more route-tracing and have higher captive juvenile mortality. Two further diet-related drivers of large AHRs are reliance on meat, and a habitat's regional primary productivity: strict carnivores have the largest AHRs, and herbivores the smallest [40,66]; while animals in resource-poor regions must range further to obtain nutrients [66,67]. If either of these underlies AHR's predictive role in welfare, then species that are naturally carnivorous, reliant on hunting (to give a more graded measure) and/or from resource-poor regions, will perform more route-tracing and have higher captive juvenile mortality.

Three final potential drivers of AHR size are predation, intra-specific population densities, and territoriality. Within non-Carnivoran mammals, highly predated species have relatively small AHRs, probably to ensure that these prey animals are familiar with local escape routes and hiding places [66]. AHRs also shrink as intra-specific population densities increase ([e.g. [66]), and as the chances of being territorial increase (large AHRs being hard to defend [66,68,69]). If similar effects hold for Carnivora, then species with naturally small AHRs might fare better in captivity because now protected from the predation or social pressures they are subject to in the wild. This hypothesis predicts that species that naturally are predated, under high social densities and/or territorial, will perform less route-tracing and have lower captive juvenile mortality.

The three potential *consequences* of being wide-ranging were as follows. One was daily travel distance: Carnivora with large AHRs naturally travel long distances daily [17,30]. A second was hippocampal volume. In primates, large species-typical AHRs correlate with large hippocampi because these improve navigational abilities [45]. The third was itinerant lifestyles: as already outlined, species with large AHRs use only small sub-regions of their annual ranges at a time before relocating. We quantified this via the ratio of daily travel distance (DD) to annual travel distance (AD) (with data on related attributes, like the number of dens used per year [17], being unavailable for most species). If any of these underlies AHR's predictive role in welfare, then species with large daily travel distances or hippocampal volumes, or with small DD:AD values, will perform more route-tracing and have higher captive juvenile mortality.

**Figure 1.**
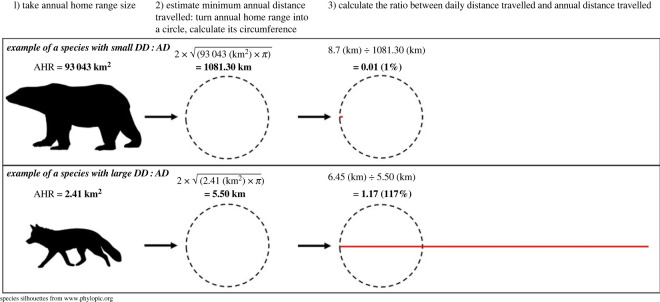
Worked calculations of unit-free daily to annual travel distance ratio (DD:AD) values, for a species with a small DD:AD value (top; polar bear) reflecting a very itinerant lifestyle, and for a sedentary species (bottom; red fox (*Vulpes vulpes*)) with a large DD:AD value. The dashed circumference of each circle (after treating all species' AHRs as this shape, for simplicity and comparability) represents a conservative estimate of minimum annual travel distance (AD). The solid red horizontal lines represent, to scale, the median distance each species travels daily (DD). The ratio of the dashed to solid line is then its DD:AD. Having a small DD:AD is one characteristic of species with large AHRs (see electronic supplementary material, table S7).

For these 12 potential correlates of AHR size, for all our 56 species, published species-level averages were used where possible (e.g. adult body mass values came from [63]), but when required, data were collated from journal articles. Electronic supplementary material, table S4 gives data collection methods, and figure 1 provides two worked examples for DD:AD. The literature was also searched to ascertain or update median AHR values (cf. methodology in [8,17,29,30,36]). Electronic supplementary material, table S5 gives all final species values for AHR, and electronic supplementary material, table S6, the final species values for the correlates of AHR.

### 2.2. Hypothesis testing

#### 2.2.1. General approach

Analyses accounted for species' shared ancestry by using a phylogenetic generalized least squares (PGLS) approach (see *Specific statistical methods* below). Two underlying assumptions were first checked: that AHR still predicts at least one measure of captive welfare; and that captive juvenile mortality reflects infant mortality (which is known to be affected by maternal stress). Once confirmed, we then proceeded with planned analyses as described next. These took a manual, stepwise approach (cf. e.g. [70]) because sample sizes and required transformations differed across variables and models, precluding an automated or Akaike information criterion (AIC)-based methodology (e.g. [71]).

We tested 12 sub-hypotheses relating to the role of the 12 potential correlates of AHR size. To do this, first, we assessed which of these did covary with AHR. Any statistically significant correlates of AHR size were also checked for collinearity (see electronic supplementary material for details). For route-tracing, we ran univariate analyses to identify which significant correlates of AHR also predicted this response to captivity. For each that did, we assessed its ability to statistically account for the AHR effect on route-tracing by investigating whether, when included in a model with AHR as a covariate, it reduced or eliminated the ability of AHR to predict route-tracing (by better accounting for the variance formerly explained by AHR; cf. [72–74]). If more than one AHR correlate significantly predicted route-tracing, even after correcting for AHR size, and these correlates were also collinear, we then assessed which correlate was the strongest or sole predictor of route-tracing (by combining them in one model).

For juvenile mortality, we took a similar approach but simply assessed univariate relationships between AHR correlates and juvenile mortality (without including AHR size as a second predictor, because AHR itself did not predict juvenile mortality: see Results). Note that all models investigating predictors of captive juvenile mortality statistically controlled for evolved species differences in life history (via the ‘age when eyes open’ altriciality metric).

For all final hypothesis-testing models, for both route-tracing and juvenile mortality, we made three further checks on the robustness of results (see *Specific statistical methods,* below), namely: running them over a ‘treeblock', checking that captive husbandry was not a confound, and looking for outlier effects.

### 2.3. Specific statistical methods

To account for shared ancestry, we used an ultrametric consensus tree for Carnivora [75] (see electronic supplementary material, figure S1). PGLS models were run using the ‘caper' R package [76] (R version 3.3.2; [77]). Following Kroshko *et al*. [30], models were only run when data were available for five or more species (for continuous predictors) or—a new refinement—five or more species per category. Residuals from PGLS models were checked for normality, data being log or square-root transformed where necessary. Where hypotheses had unidirectional predictions, we used one-tailed tests to increase power [78,79]. To assess and compare model fit, *adjusted R*^2^s are presented to correct for the number of terms in a model (e.g. [80]).

All final significant hypothesis-testing models were then subject to three checks for robustness: (i) being re-run over a treeblock to account for phylogenetic uncertainty; (ii) assessment of the role of species differences in captive husbandry; and (iii) checks to assess whether any effects strongly depended on the leverage of particular individual species (e.g. outliers). Only the latter ever changed results, and so is described here (with the electronic supplementary material giving further details). To assess whether any results relied on specific species, we used a custom version of the influ_phylm function within the ‘sensiPhy' R package [81]. This performs ‘leave-one-out' deletion analyses by removing each species in turn, the package deeming species ‘influential' if removing it resulted in a standardized difference greater than 2 in parameter estimates. When such species were removed, the model was re-run, and the slope and corresponding *p* value recalculated for each term to assess the impact of these influential data points on results.

## 3. Results

The check to confirm the original home range effect showed that, using our updated values, individuals from naturally wide-ranging species did spend significantly more time route-tracing (*t* = 2.45, *F*_1, 21_ = 5.98, *N* = 23, *R*^2^ = 0.18, *λ* = 0.52, *p* = 0.01). AHR did not, however, predict juvenile mortality (*t* = 0.22, *F*_1, 31_ = 0.05, *N* = 33, *R*^2^ = −0.03, *λ* = 0.02, *p* = 0.41), even when altriciality was controlled for (whole model: *F*_2, 26_ = 0.22, *N* = 29, *R*^2^ = −0.06, *λ* = 0, *p* = 0.80; AHR term: *t* = 0.53, *p* = 0.30). Juvenile mortality values were also predicted by infant mortality (see electronic supplementary material).

### 3.1. Investigating correlates of annual home range size and their impact on welfare

When tested, four of the 12 potential correlates of AHR were rejected as such (group metabolic need, trophic level, percentage of hunted meat in diet [i.e. live prey, both vertebrate and invertebrate], and hippocampal volume; see electronic supplementary material, table S7), and so were dropped from subsequent analyses. Of the remaining eight potential correlates of AHR that were confirmed as such, some were collinear (see electronic supplementary material, table S8).

### 3.2. Predictors of route-tracing

**Figure 2.**
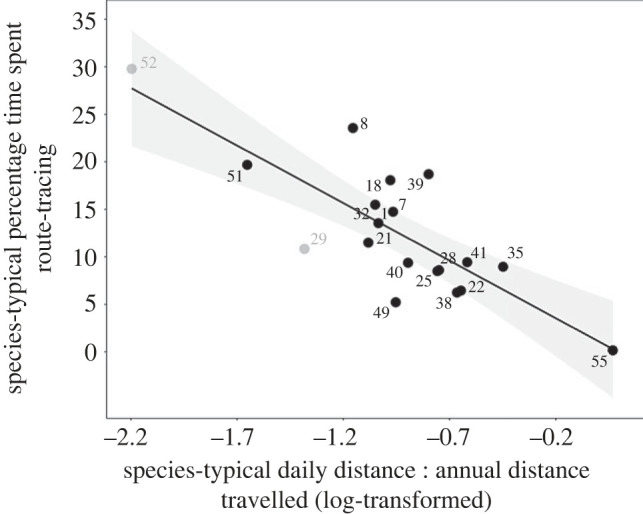
Ratio of daily : annual distances (DD:AD) travelled predicts time spent route-tracing. Species that naturally cover only small proportions of their estimated annual travel distances each day (see electronic supplementary material, table S4 for calculations; DD:AD) develop the most time-consuming route-tracing. The regression line accounts for phylogenetic relatedness; shading shows the 95% CI. Each point represents a species: 1, cheetah (*Acinonyx jubatus*); 7, caracal (*Caracal caracal*); 8, Asiatic golden cat (*Catopuma temminckii*); 18, sun bear (*Helarctos malayanus*); 21, Geoffroy's cat (*Leopardus geoffroyi*); 22, ocelot (*Leopardus pardalis*); 25, serval (*Leptailurus serval*); 28, Canadian lynx (*Lynx canadensis*); 29, Eurasian lynx (*Lynx lynx*); 32, sloth bear (*Melursus ursinus*); 35, American mink (*Neovison vison*); 38, lion; 39, jaguar (*Panthera onca*); 40, leopard (*Panthera pardus*); 41, tiger (*Panthera tigris*); 49, snow leopard (*Uncia uncia*); 51, brown bear (*Ursus arctos*); 52, polar bear; 55, red fox. Checks identified species (29) and (52) as ‘influential' (shown in grey), but removing neither changed the overall effect (see main text and electronic supplementary material, table S9).

Univariate analyses investigating which of these eight AHR correlates individually predicted route-tracing, showed that daily distance travelled and habitat productivity did not (daily distance travelled: *t* = 0.29, *F*_1, 17_ = 0.09, *N* = 19, *R*^2^ = −0.05, *λ* = 0.61, *p* = 0.39; habitat productivity: *t* = 1.46, *F*_1, 22_ = 2.14, *N* = 24, *R*^2^ = 0.05, *λ* = 0.49, *p* = 0.16), while territoriality could not be investigated (only two species being non-territorial). The remaining five AHR correlates did predict route-tracing, or at least tended to (table 1), the strongest effect being that itinerant species were most stereotypic (figure 2).

**Table 1.** Correlates of annual home range (AHR) size in Carnivora that predict route-tracing in univariate analyses. Significant terms (*p* < 0.05) are shown in bold, trends (*p* ≤ 0.1) in italics.

correlate of annual AHR size	relationship with route-tracing
*body mass*	*t* *=* *1.39, F*_1, 22_ *=* *1.93, N* *=* *24, R^2^* *=* *0.04, λ* *=* *0.95, p* *=* *0.09*
**individual metabolic need**	***t* = 2.25, *F*_1, 20_ = 5.06, *N* = 22, *R*^2^ = 0.16, *λ* = 0.96, *p* = 0.02**
*population density*	*t* *=* *−1.32, F*_1, 15_ *=* *1.74, N* *=* *17, R^2^* *=* *0.04, λ* *=* *0.98, p* *=* *0.10*
**predation pressure**	***t* = −2.30, *F*_1, 16_ = 5.28, *N* = 18, *R*^2^ = 0.20, *λ* = 0.40, *p* = 0.02**
**daily : annual distances travelled**	***t* = −5.59, *F*_1, 17_ = 31.22, *N* = 19, *R*^2^ = 0.63, *λ* = 0, *p* < 0.0001**

Next, each of these five variables was added as a covariate into models with AHR size. Here, none statistically eliminated the AHR effect on route-tracing (table 2), but three—body mass, individual metabolic need and natural population density—ceased themselves to predict route-tracing, showing that their previous apparent effects were just by-products of their relationships with AHR. The other two—predation risk and DD:AD—remained predictive once AHR size was controlled for. However, these variables were collinear: predated species cover more of their annual travel distance each day than do non-predated (see electronic supplementary material, table S8). Including both variables in one model, to identify which was truly predictive of route-tracing, gave the following: for predation risk: *t* = −0.08, *p* = 0.94; for DD:AD: *t* = −3.19, *p* = 0.01; and for AHR size: *t* = 2.38, *p* = 0.02 (whole model: *F*_3, 9_ = 9.53, *N* = 13, *λ* = 0.13, *R*^2^ = 0.68, *p* < 0.01). The apparent effect of predation risk on route-tracing was thus just a by-product of its relationship with DD:AD; while species that cover only small fractions of their annual ranges daily are significantly more prone to route-tracing (AHR size also seeming to remain predictive).

**Table 2.** Correlates of annual home range (AHR) size, and their relationships with route-tracing when AHR is included as a second predictor variable (significant terms shown in italics). For these correlates' definitions and methods of calculation, see electronic supplementary material, table S4.

correlate of AHR size ('correlate' for short)	relationship between AHR and route-tracing with correlate added to the model	relationship between correlate and route-tracing	whole model *F*, *N*, *R*^2^, *λ*, and *p*
body mass	*t = 1.98, p = 0.03*	*t* = −0.06, *p* = 0.95	*F*_2, 18_ = 2.58, *N* = 21, *R^2^* = 0.14, *λ* = 0.84, *p* = 0.10
individual metabolic need	*t = 2.06, p = 0.03*	*t* = 0.15, *p* = 0.44	*F*_2, 16_ = 2.95, *N* = 19, *R^2^* = 0.18, *λ* = 0.71, *p* = 0.08
population density	*t = 1.97, p = 0.04*	*t* = −0.02, *p* = 0.49	*F*_2, 13_ = 4.16, *N* = 16, *R*^2^ = 0.30, *λ* = 0.80, *p* = 0.04
predation risk^a^ (adults typically predated versus not predated)	*t = 2.23, p = 0.02*	*t = −1.78, p = 0.05 (predated species route-trace less)*	*F*_2, 14_ = 6.22, *N* = 17, *R*^2^ = 0.40, *λ* = 0.42, *p* = 0.01
daily : annual travel distances (DD:AD)^b^	*t = 2.04, p = 0.03^b^*	*t = −2.20, p = 0.02*	*F*_2, 16_ = 7.62, *N* = 19, *R*^2^ = 0.42, *λ* = 0.30, *p* < 0.01

^a^These two variables were collinear; and when both were combined in a model predicting route-tracing, only DD:AD had an effect (see text).

^b^Subsequent checks showed that this apparent effect was driven by an influential species: the polar bear (see text and electronic supplementary material, table S9).

The robustness of this final result was then checked. ‘Leave-one-out' analyses first investigated the model analysing combined effects of DD:AD and AHR on route-tracing (see electronic supplementary material, table S9). For the DD:AD term, the red fox was flagged as influential. However, even without this species, DD:AD still negatively predicted route-tracing (see electronic supplementary material, table S9; whole model: *F*_2, 15_ = 5.22, *N* = 18, *R*^2^ = 0.33, *λ* = 0.28, *p* = 0.02; DD:AD term: *t* = −1.70, *p* = 0.05, AHR term: *t* = 1.35, *p* = 0.10). The other farmed species (American mink) was not influential. For the AHR term, the polar bear was flagged as influential (see electronic supplementary material, table S9), and removing this rendered the AHR effect non-significant, although DD:AD remained predictive (whole model: *F*_2, 15_ = 4.60, *N* = 18, *R*^2^ = 0.30, *λ* = 0.09, *p* = 0.03; DD:AD term: *t* = −2.57, *p* = 0.01, AHR term: *t* = 0.22, *p* = 0.41). DD:AD was therefore a robust predictor of route-tracing, while in contrast AHR size had no independent effect on route-tracing (beyond the leverage of one extreme species). Thus the previous apparent effect of AHR on route-tracing was driven by DD:AD, since the latter's inclusion statistically eliminates the AHR effect. (Please see electronic supplementary material for extra details and checks).

‘Leave-one-out' analyses were then applied to the model investigating the effect of DD:AD alone on route-tracing (cf. figure 2). This again identified the polar bear as influential, as well as the Eurasian lynx, but neither changed the direction or significance of the DD:AD effect when removed (without polar bear: *R*^2^ = 0.44, *p* < 0.0001; without Eurasian lynx: *R*^2^ = 0.69, *p* < 0.0001; see electronic supplementary material, table S9), so confirming this result's robustness.

### 3.3. Captive juvenile mortality

**Figure 3.**
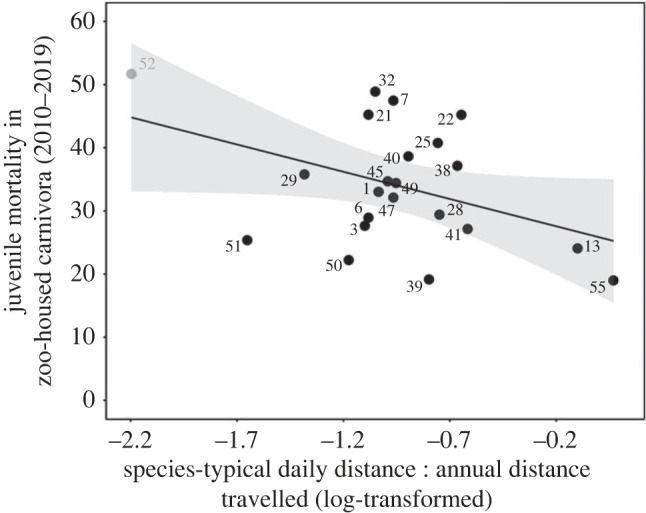
Ratio of daily : annual distances travelled predicts captive juvenile mortality rates. Species that naturally cover only small proportions of their estimated annual travel distances each day (DD:AD) have the highest juvenile mortality in captivity. The regression line accounts for phylogenetic relatedness, and shading shows the 95% CI. Each point represents a species, cf. legend for figure 2 plus: 3, red panda (*Ailurus fulgens*); 6, wolf (*Canis lupus*); 13, black-footed cat (*Felis nigripes*); 45, leopard cat (*Prionailurus bengalensis*); 47, cougar (*Puma concolor*); 50, American black bear (*Ursus americanus*). The polar bear (52) was identified as ‘influential', shown in grey, but removal did not change the overall effect (see text and electronic supplementary material, table S9). The effect was also maintained after controlling for offspring altriciality (see text).

Of the eight correlates of AHR, only DD:AD significantly predicted juvenile deaths (*t* = −2.04, *F*_1, 20_ = 4.18, *N* = 22, *R*^2^ = 0.13, *λ* = 0, *p* = 0.03; figure 3), an effect remaining even after controlling for offspring altriciality (whole model: *F*_2, 18_ = 3.53, *N* = 21, *R*^2^ = 0.20, *λ* = 0, *p* = 0.05; DD:AD term: *t* = −2.66, *p* = 0.01, partial *R*^2^ for DD:AD = 0.28). (See electronic supplementary material, table S10 for full details and non-significant relationships with the other seven correlates.) ‘Leave-one-out' checks for robustness analyses again flagged polar bears as influential, but excluding this species did not alter the interpretation of the result (see electronic supplementary material, table S9; whole model: *F*_2, 17_ = 2.33, *N* = 20, *R*^2^ = 0.12, *λ* = 0, *p* = 0.13; DD:AD term: *t* = −1.77, *p* = 0.05; partial *R*^2^ for DD:AD = 0.16). Thus, DD:AD was a robust predictor of captive juvenile losses, with naturally more itinerant species faring worse.

## 4. Discussion

This investigation into why wide-ranging Carnivora tend to struggle to adjust to captivity, thus identified naturally itinerant lifestyles as the critical predictor of their captive welfare. This new finding helps pinpoint important mismatches between captivity and the wild conditions in which these animals evolved, so advancing the understanding of species differences in Carnivore welfare. It also has practical implications for animal care and captive breeding, and suggests novel hypotheses for future work. First, our results (electronic supplementary material, table S1) confirmed that there is considerable inter-species variation in how Carnivora react to captivity, even in today's modern zoos and aquaria. Some show little stereotypic behaviour, and also have low captive juvenile losses (e.g. red and Arctic foxes, plus American black bears, all lose under 26% zoo-born cubs before 1 year of age: in the bottom quintile of our dataset, and with lower mortality than in the wild (which is 39–76% for red fox cubs, as one example; cf. e.g. [82–85]). Yet in other species, individuals spend hours per day route-tracing, and, despite being protected from disease, starvation and predation, captive infants and juveniles have low survival. For example, grey seals (*Halichoerus grypus*) show very time-consuming route-tracing, and lose 63% of zoo-born pups before the age of one: the highest juvenile mortality in our dataset, and also worse than the *ca* 40–60% mortality seen in the wild [86,87]. Responses to captivity like these are ethically concerning. When captive breeding aims to aid conservation, they are practically concerning too, reducing the effective sizes of already small populations, compromising sustainability and warning of intense new domestication-like selection pressures [2]. Polar bears, for instance, are classed by the IUCN as Vulnerable [6], yet are not self-sustaining in zoos [58]; route-tracing bears spend a median of 52% of observations doing so; and under half of captive-born cubs reach the age of 1 (their mortality values of 51.7% being in the top quintile of our dataset, and seemingly higher than the 45% estimated in the wild [88]).

What prevents such species from coping as well as more successful ones? Previous research identified large annual home ranges (AHRs) as a welfare risk factor, and/or small AHRs as welfare-protective, yet this could not explain the majority of inter-species variation in captive welfare, nor specify what it is about natural ranging behaviour that underlies this effect. By systematically investigating a range of potential correlates of AHR size (evolutionary drivers and behavioural/cognitive consequences), our current analyses show that several potential explanations for this effect are *not* influential. Wide-ranging Carnivora are thus not prone to poor captive welfare because they travel relatively long distances each day in the wild; are larger bodied and more carnivorous, with higher metabolic needs; or naturally live at low social densities. Furthermore, nor are species with small annual home ranges pre-adapted to captivity because it spares them from the predation they are naturally subject to, or from the territorial defence they perform in the wild. Instead, welfare outcomes were predicted by our metric of itinerant lifestyles, ‘DD:AD’—the ratio of daily travel distance to a conservative estimate of minimum annual travel distance. For route-tracing, DD:AD thus explained at least 44% of inter-species variation (excluding the influential polar bear; 69% excluding the influential Eurasian lynx; and 53% if both were excluded). Furthermore, DD:AD fully explained the AHR effect on this stereotypic behaviour (once polar bears were removed for being unduly influential): it statistically eliminated the ability of AHR to predict route-tracing, by better accounting for the variance formerly explained by this term. For captive juvenile mortality, DD:AD also explained 16% of inter-species variation (after excluding the influential polar bear), while AHR had no significant effects. Together, this shows that the previous weak, somewhat inconsistent, apparent effects of natural home range size on welfare were driven by the underlying effect of this correlated, stronger influence of naturally itinerant lifestyles.

Naturally roaming Carnivora—semi-nomadic animals that relocate multiple times a year, covering just small fractions of their ranges in an average day—are thus most prone to welfare problems in captivity (with annual range size *per se* having no independent effects): a finding that generates new predictions about further at-risk or protected species, and new hypotheses about further adverse responses to captivity. For Carnivora lacking complete welfare data in our current dataset, for instance, it indicates that coyotes (*Canis latrans*) should fare well in captivity (with their high DD:AD of 0.86; see electronic supplementary material, table S6); while the closely related wolf with its low DD:AD (0.08), should be prone to more severe route-tracing and higher juvenile mortality. It also predicts similar problems in giant pandas (*Ailuopoda melanopoda),* which have one of the smallest DD:AD values in our dataset (0.04): despite a small median home range (just 7.6 km^2^, or 2.83 km in circumference), giant pandas typically use a tiny fraction of this daily (shifting between sites to e.g. follow fluctuating bamboo protein levels; [89]). And perhaps this naturally roaming lifestyle also explains why achieving live captive-bred young is so challenging for this species, since conception rates and prepartum losses are also sensitive to stress [90]. Indeed, as previously noted, across 15 species including giant pandas, large AHRs have already been found to predict low offspring production, albeit only weakly (*R*^2^ = 0.25). Our results suggest that DD:AD would prove a much stronger predictor of this captive fertility metric. Turning to abilities to adjust to more restrictive captive conditions, of the diverse wild Carnivora that farmers have attempted to breed for fur (including Canadian lynx and various martens [91]), it is striking that today's most commonly farmed species are red foxes and American mink*,* both with large DD:AD ratios (1.17 and 0.36, respectively). To cope with agricultural usage like this, naturally small home ranges have long been proposed as favourable species traits (e.g. [3]). Our results now suggest an alternative hypothesis: that naturally sedentary lifestyles, in which animals spend all their time in one ‘extremely familiar, intimately known patch of land’ [17], are the key.

Testing such hypotheses thus generates broad cross-species principles. These cannot explain *all* variation in welfare, such as that arising from individual differences in captive care, or from the idiosyncrasies of particular species (e.g. those we identified as ‘influential’). However, they can reveal taxon-wide patterns that are fundamentally fascinating, and also practically useful too. Such principles can inform strategic collection planning, for instance, by indicating types of animal that are intrinsically more challenging to keep successfully, such that some enterprises might ideally triage them out. To illustrate why this is useful, zoos currently stretch their resources across some 4000 species, which then constrains the sizes of many of their global populations, even for Threatened species (e.g. to under 100 individuals, as for 115 of the 173 Carnivore species in Species360 institutions [60]). To achieve large, self-sustaining, low-stress populations, it might be rational to phase out struggling species, and instead build up larger populations of those that are inherently more likely to flourish. But if some naturally itinerant species *are* still to be kept in captivity (e.g. giant pandas and polar bears), then a second practical use of these cross-species principles is to tailor captive care to better meet their needs. While our results cannot *definitively* pinpoint the precise aspects of captivity that most frustrate these animals, they do indicate that providing more space *per se*, opportunities to locomote (e.g. running wheels), and hunting opportunities or foraging ‘enrichments', will *not* tackle these animals' root problems (perhaps why meta-analyses suggest that the latter approach, while commonly used, on average only reduces route-tracing by about half [92]). Furthermore, the characteristics of naturally itinerant lifestyles usefully suggest three alternative strategies as more likely to be effective: providing more opportunities for control, more novelty and/or cognitive challenge, each addressed below.

Having more *control* could be critical because itinerant lifestyles involve regularly choosing between options, and making decisions, not least about whether to stay or go: thus when to leave a given site for a new one, in what direction to travel, and when and where to re-settle [30,42,44,89]. Indeed, stereotypic route-tracing could represent captive Carnivores' frustrated attempts to relocate in this way. Welfare might then best be improved by giving such subjects more agency, including about where to live, e.g. allowing choices between multiple locations within a larger exhibit (e.g. via interconnected enclosures, constant access to on- and off-exhibit areas [22,23,93,94], and multiple dens [30]). Animals could also be given control over access to preferred stimuli like food, and their exposure to warmth, ventilation, showers, light levels, the sight of humans and so on, perhaps via creative IT technologies (e.g. [95]). *Novelty* could instead be particularly important, because itinerant lifestyles both reflect naturally variable environments (e.g. [44]), and also expose animals to unfamiliar stimuli as they shift between regions (e.g. [42]). Optimal strategies for improving welfare should then focus on mitigating boredom (e.g. [96]), for instance, by rotating animals between different enclosures (e.g. [22,23]), using naturalistic exhibits that change with season (e.g. [49]), providing stimulating views (e.g. [97]) and regularly introducing unfamiliar stimuli. Finally, itinerant species may need more *cognitive challenge*, because in the wild they must integrate and weigh up diverse forms of information, learn when and where to find key resources, and build up cognitive maps to support navigation (e.g. [42,98]). If this is most important, then welfare will best be improved by ‘cognitive enrichments' that provide stimulating problems to solve (e.g. [99]); by the learning opportunities offered by training (cf. e.g. [97,100]); and perhaps even by complex mazes designed to create spatial challenges [101].

Some zoos are already trying these three approaches, to incorporate aspects of ranging (e.g. those studies cited above, and also [102]). Which works best has not yet been assessed, but doing so, via new meta-analyses or via large-scale controlled experiments, would be an exciting next step. In parallel, future species comparisons could seek to specify the crucial attributes of itinerant species, improving yet further the understanding of at-risk Carnivora. These should aim to rectify some shortcomings in the current datasets (largely reflecting the limitations of meta-analysing data from published zoo studies; see electronic supplementary material for details). They could then also take three new, complementary approaches. One is to further investigate cognitive or neurological traits as potential welfare predictors (such as neophilia; hippocampal volume, re-assessed with bigger sample sizes [see electronic supplementary material]; and cage-side measures of hippocampal function; cf. [103]). The second is to use the tracking expertise of movement ecologists (cf. e.g. [104]), to assess specific aspects of itinerant lifestyles that might better predict welfare (e.g. the number of dens used annually; number of habitat types encountered a year; average residence times per location; and how often animals revisit sites within their range). A third, once the dataset is large enough to permit this, is to investigate sex and subpopulation effects (for instance, to capture how males and females often have different AHR sizes, reflecting their different reproductive strategies; see electronic supplementary material for details).

Together, answers would further help improve captive breeding and welfare, perhaps even leading to the ‘…global reduction of stereotypies' called for by Roller *et al*. [38]. They could shed light on fascinating, fundamental ‘evo-mecho’ topics (such as which ecological niches make it adaptive for animals to prefer choice and control, or favour the evolution of strong motivations to explore). Answers might even speak to the provocative question posed by Tidière *et al*. [105]: ‘Do animals, even when born and raised in zoos, perceive their enclosures as a spatial constraint in terms of compressed home ranges, or as an actual restriction of freedom in terms of a limitation of their own choices?' Pending such research, the present findings advance our understanding of evolutionary bases for welfare problems in captive Carnivora, showing that naturally itinerant lifestyles, rather than large annual ranges *per se*, confer the greatest risk. We hope this assists collection management decisions, and inspires yet more innovations by zoos to improve Carnivore well-being.

## Data Availability

Data available from the Dryad Digital Repository: https://doi.org/doi:10.5061/dryad.pk0p2ngsp [106]. The data are provided in electronic supplementary material [107].
