# Correction: ‘The welfare problems of wide-ranging Carnivora reflect naturally itinerant lifestyles’ (2023), by Bandeli *et al.*

**DOI:** 10.1098/rsos.231509

**Published:** 2023-12-06

**Authors:** Georgia J. Mason

**Keywords:** *welfare*, captivity, zoos, choice, control, Carnivora

*R. Soc. Open Sci.* **10**: 230437 (Published online 6 September 2023). (https://doi.org/10.1098/rsos.230437)

Corrigendum to the last five papers in the reference list of Bandeli *et al*. [1]: reference [101] (Veasey 2020) was accidentally omitted in the reference list, which in turn meant that three of the last few references in the list [101, 103, 104] were mis-numbered with respect to the text.

With Veasey 2020 re-inserted (and two other references swapped in position) the correct numbering for the last few references (now [101–105]) is below:

101. Veasey JS. 2020 Can zoos ever be big enough for large wild animals? A review using an Expert Panel Assessment of the psychological priorities of the Amur tiger (*Panthera tigris altaica*) as a model species. *Animals* **10**, 1536. (doi:10.3390/ani10091536)

102. Kirchgessner ML, Sewall BJ. 2015 The impact of environmental, social, and animal factors on visitor stay times at big cat exhibits. *Visitor Studies* **18**, 150–167. (doi:10.1080/10645578.2015.1079091)

103. Cahusac PM, Miyashita Y, Rolls ET. 1989 Responses of hippocampal formation neurons in the monkey related to delayed spatial response and object-place memory tasks. *Behavioural Brain Research* **33**, 229–240. (doi:10.1016/S0166-4328(89)80118-4)

104. Abrahms B *et al.* 2017 Suite of simple metrics reveals common movement syndromes across vertebrate taxa. *Movement Ecology* **5**, 12. (doi:10.1186/s40462-017-0104-2)

105. Tidière M, Gaillard JM, Berger V, Müller DW, Bingaman Lackey L, Gimenez O, Clauss M, Lemaître JF. 2016 Comparative analyses of longevity and senescence reveal variable survival benefits of living in zoos across mammals. *Scientific Reports* **6**, 36361. (doi:10.1038/srep36361)

(Plus 106 and 107 now refer to supporting/archived online material)

In the text, this means that in the final two paragraphs of the Discussion, reference [101] should be [103], reference [103] should be [104], and reference [104] should be [105].

This has been corrected on the publisher's website.

## References

[1] Bandeli M, Mellor EL, Kroshko J, Maherali H, Mason GJ. 2023 The welfare problems of wide-ranging Carnivora reflect naturally itinerant lifestyles. R. Soc. Open Sci. **10**, 230437. (10.1098/rsos.230437) 37680500 PMC10480699

